# Left hemicolectomy and low anterior resection in colorectal cancer patients: Knight–griffen vs. transanal purse-string suture anastomosis with no-coil placement

**DOI:** 10.3389/fsurg.2023.1093347

**Published:** 2023-04-17

**Authors:** Michele Ammendola, Francesco Filice, Caterina Battaglia, Roberto Romano, Francesco Manti, Roberto Minici, Nicola de'Angelis, Riccardo Memeo, Domenico Laganà, Giuseppe Navarra, Severino Montemurro, Giuseppe Currò

**Affiliations:** ^1^ Science of Health Department, Digestive Surgery Unit, University “Magna Graecia” Medical School, Catanzaro, Italy; ^2^ Radiology Division, Department of Experimental and Clinical Medicine, University Hospital Mater Domini, “Magna Graecia” University of Catanzaro, Catanzaro, Italy; ^3^ Unit of Colorectal and Digestive Surgery, DIGEST Department, Beaujon University Hospital (AP-HP), University Paris Cité, Clichy, France; ^4^ Hepato-Biliary and Pancreatic Surgical Unit, “F. Miulli” Hospital, Acquaviva Delle Fonti, Bari, Italy; ^5^ Department of Human Pathology of Adult and Evolutive Age, Surgical Oncology Division, “G. Martino” Hospital, University of Messina, Messina, Italy; ^6^ Science of Health Department, General Surgery Unit, University “Magna Graecia” Medical School, Catanzaro, Italy

**Keywords:** colorectal cancer, colorectal anastomosis, complications, no-Coil, surgical oncology

## Abstract

**Background:**

Colorectal cancer (CRC) is considered one of the most frequent neoplasms of the digestive tract with a high mortality rate. Left hemicolectomy (LC) and low anterior resection (LAR) with minimally invasive laparoscopic and robotic approaches or with the open technique are the gold standard curative treatment.

**Materials and methods:**

Seventy-seven patients diagnosed with CRC were recruited between September 2017 and September 2021. All patients underwent a preoperative staging with a full-body CT scan. The goal of this study was to compare both types of surgeries, LC-LAR LS with Knight–Griffen colorectal anastomosis and LC-LAR open with Trans-Anal Purse-String Suture Anastomosis (the TAPSSA group), by positioning a No-Coil transanal tube (SapiMed Spa, Alessandria, Italy), in terms of postoperative complications such as prolonged postoperative ileus (PPOI), anastomotic leak (AL), postoperative ileus (POI), and hospital stay.

**Results:**

The patients were divided into two groups: the first with 39 patients who underwent LC and LAR in LS with Knight–Griffen anastomosis (Knight–Griffen group) and the second with 38 patients who underwent LC and LAR by the open technique with the TAPSSA group. Only one patient who underwent the open technique suffered AL. POI was 3.76 ± 1.7 days in the TAPSSA group and 3.07 ± 1.3 days in the Knight–Griffen group. There were no statistically significant differences in terms of AL and POI between the two different groups.

**Conclusion:**

The important point that preliminarily emerged from this retrospective study was that the two different techniques showed similarities in terms of AL and POI, and therefore, all the advantages reported in the previous studies pertaining to No-Coil also hold good in this study regardless of the surgical technique used. However, randomized controlled trials are needed to confirm these findings.

## Introduction

**Figure 1:**
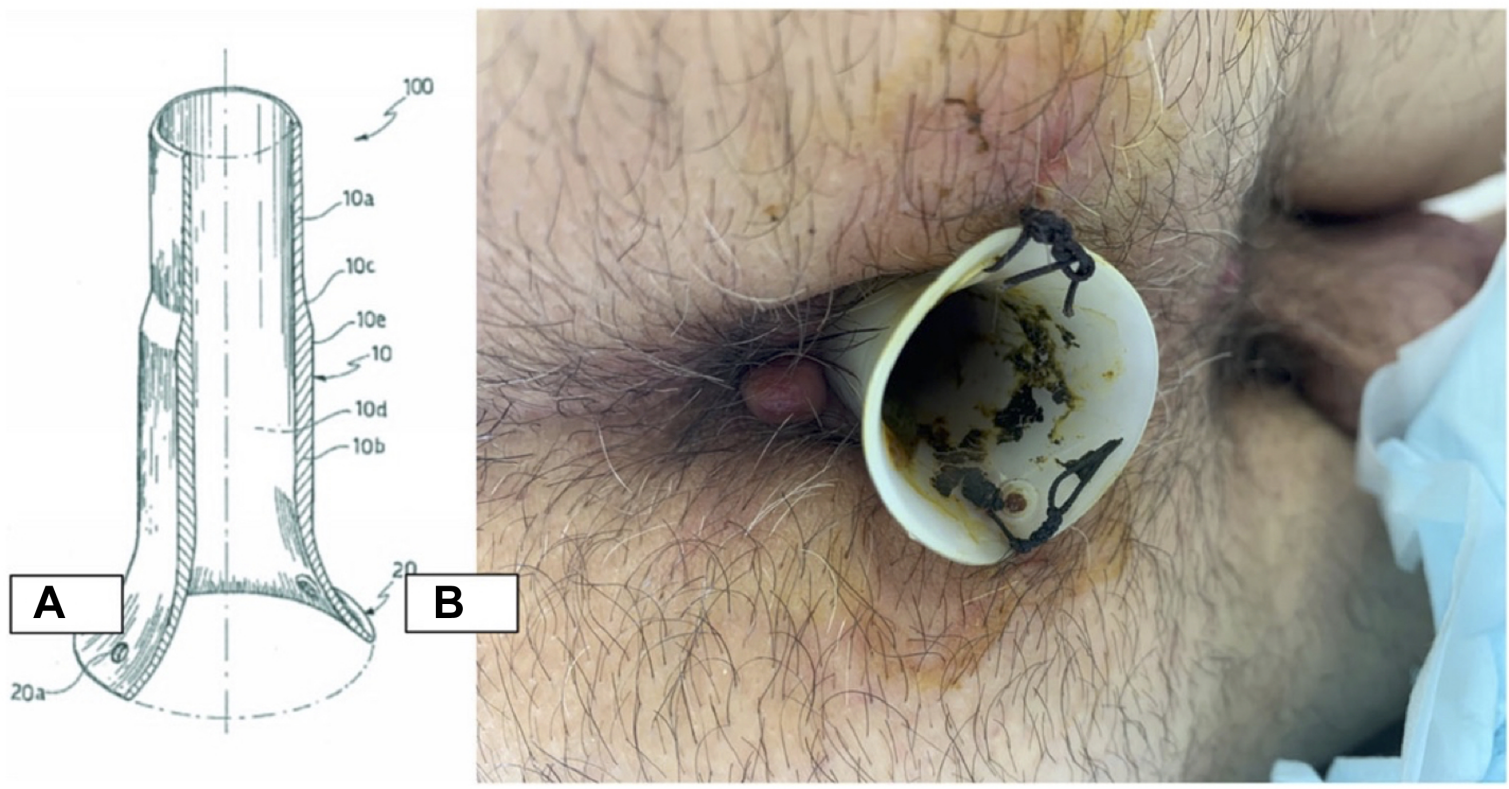
No coil structure and postoperative placement. (**A**) Length of 60–80 mm, thickness of 2 mm, and diameter of 20 mm. (**B**) Stabilized 6–8 cm far from the anus through two stitches.

Colorectal cancer (CRC) is considered the most common malignancy in Western countries (1). Accurate preoperative staging is crucial for planning the optimal therapeutic strategy for individual patients. In the preoperative staging of colorectal cancer, computed tomography (CT) is often necessary for devising the best plan for surgery and/or neoadjuvant therapy, particularly when local tumor extension into adjacent organs or distant metastases is detected (2). Low anterior resection (LAR), left hemicolectomy (LC), and right hemicolectomy (RC) remain the treatments of choice, ensuring the best results in terms of quality of life (QoL) and overall survival (Os) (3). It is possible to perform this type of surgery with minimally invasive laparoscopic (LS) and robotic approaches or with the open technique (4). All types of surgeries may cause different complications, and the most frequent of these are prolonged postoperative ileus (PPOI) and anastomotic leak (AL) (5–7). Several factors may contribute to PPOI occurrence, but the level of anastomosis detected is probably the most important one. The parameters used in the literature to describe the incidence rate of AL, regardless of the technicalities surrounding stapled or hand-sewn anastomoses, are at complete variance with the ones used presently, and these depend on the nature of the site in question, that is, a rate of up to 6% for ileo-colic anastomoses, up to 9% for colo-colonic anastomoses, and up to 20% for colo-rectal anastomoses (6). Different studies demonstrate that increased intraluminal rectal pressure is the major contributor to AL (8–11), and for this reason, different endorectal devices (e.g., transanal tube cuff rectum, drainage tube, and silicone transanal tube) have been proposed as promising alternatives to stoma (12, 13). No-Coil® is a transanal silicone stent that allows endorectal decompression, and it is used for the anastomosis of the lower gastrointestinal tract (Figure 1) (14, 15).

According to recent studies, No-Coil could be considered a useful tool in the prevention of AL-related complications, as it is characterized by ease of use and good feasibility, cost-effectiveness, and favorable patient quality-of-life outcomes after treatment (16). Nevertheless, evidence about No-Coil implementation in the surgical treatment of CRC is limited to a few studies, and definitive conclusions in terms of efficacy cannot be made. Furthermore, these studies examined No-Coil by following the LAR approach, and evidence about its efficacy by following the LC approach is scarce (17).

**Figure 2:**
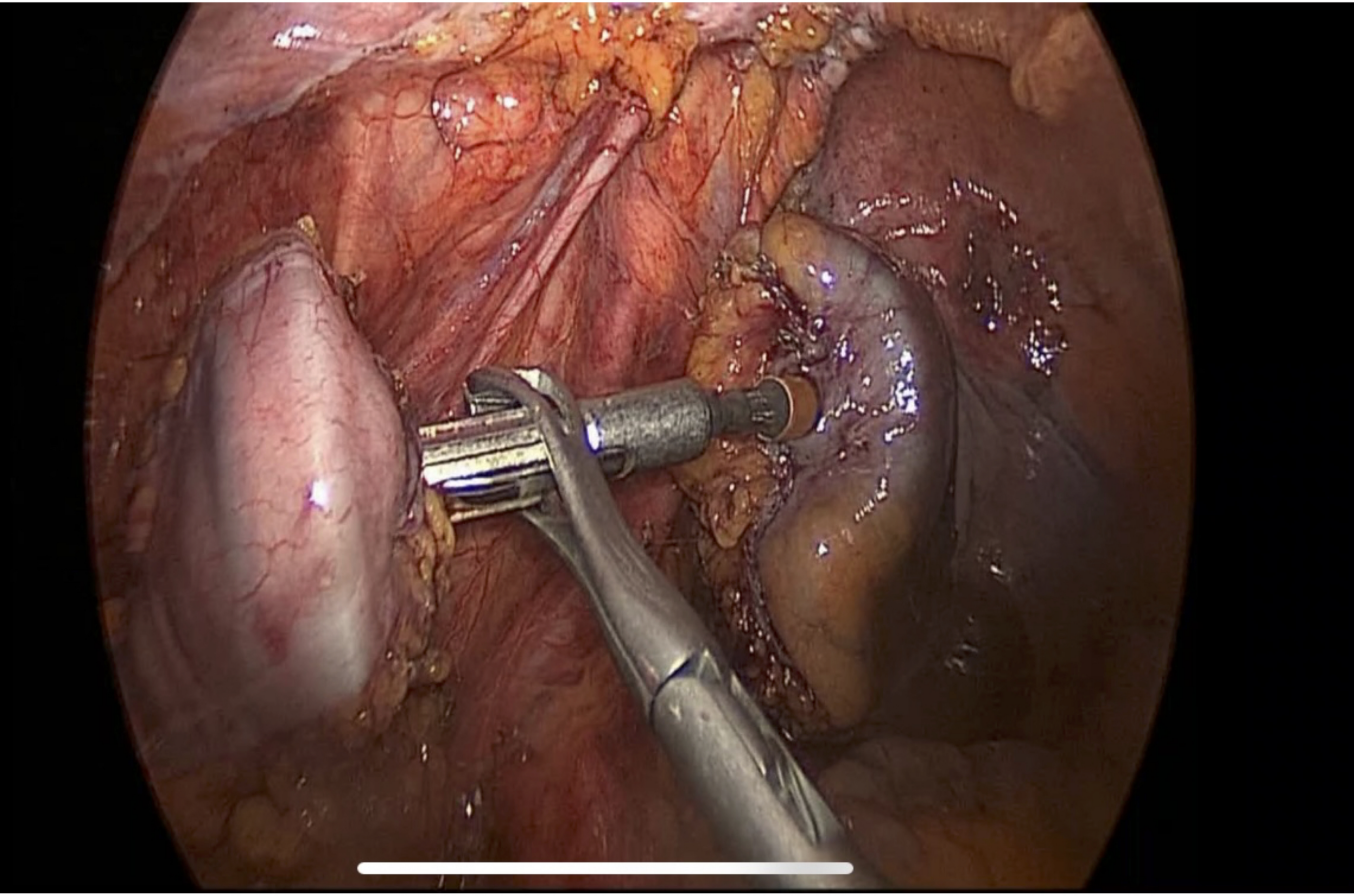
Knight–Griffen colorectal anastomosis.

**Figure 3:**
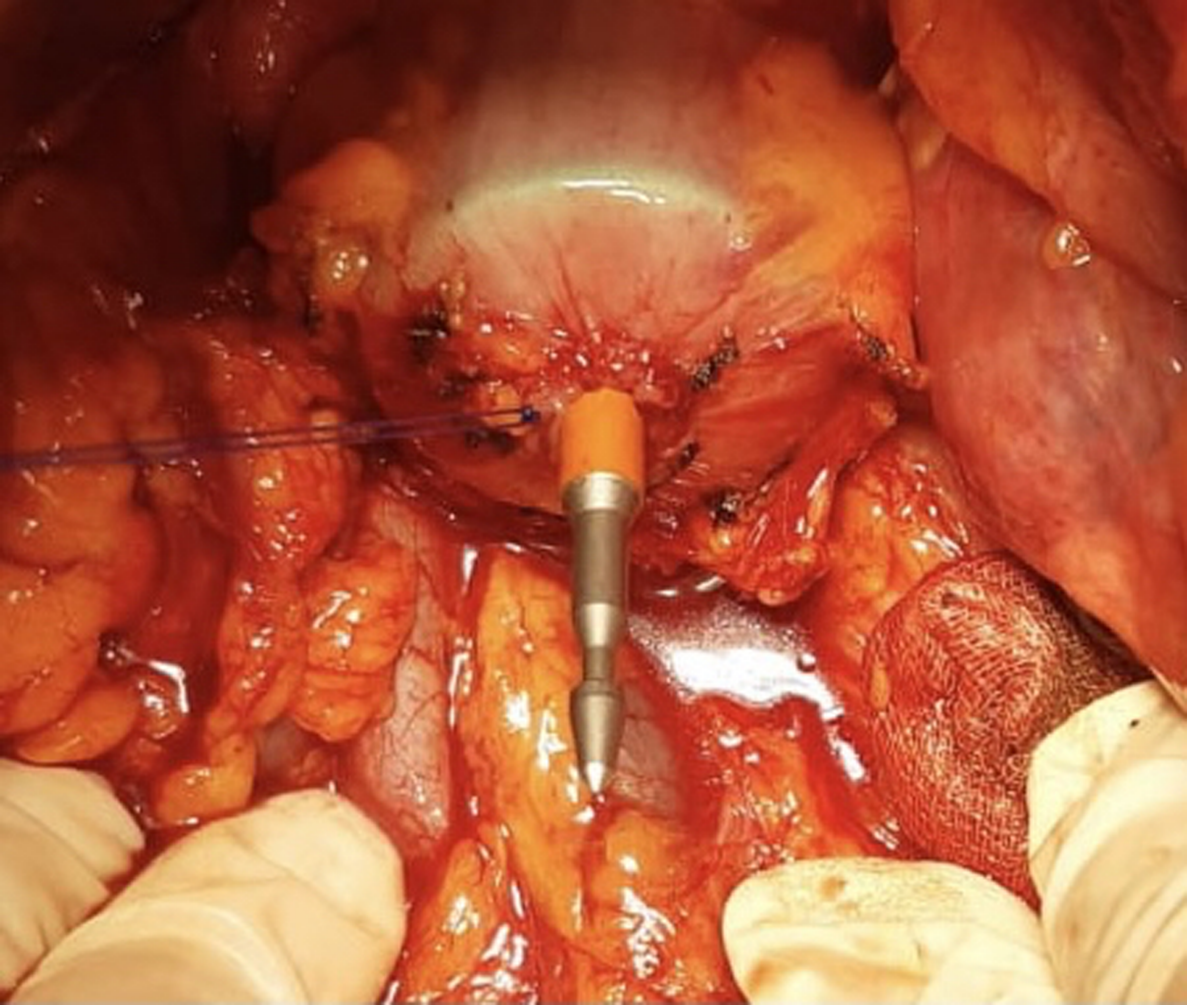
Transanal purse-string suture anastomosis.

The aim of this study is to improve and analyze the evidence concerning the use of No-Coil and verify its results in two different types of surgeries: LC-LAR LS with Knight–Griffen colorectal anastomosis (Figure 2) and LC-LAR by the open technique with Trans-Anal Purse-String Suture Anastomosis (Figure 3). We analyzed the period of postsurgical hospitalization and the presence of flatus or other complications in all patients who underwent laparoscopic (LS) LAR/LC and open LAR/LC with a No-Coil transanal tube placed at the end of the surgical procedure.

## Materials and methods

A retrospective study was conducted between September 2017 and September 2021 in which 77 patients diagnosed with a CRC of the descending colon, splenic flexure, sigma, and rectum were recruited at the Digestive Surgery Unit of “Magna Graecia” University Medical School, “Mater Domini” Hospital. LC-LAR LS with Knight–Griffen colorectal anastomosis was performed in 39 patients. Meanwhile, 38 patients underwent LC and LAR by the open technique with Trans-Anal Purse-String Suture Anastomosis (TAPSSA) on Circular Stapler Circular by both techniques measuring 33 mm. All interventions were performed by the same surgeon (MA) in order to reduce possible interoperator errors and distortions and to achieve better accuracy. LC was performed for sigmoid and descending colon tumors, while LAR was applied for the neoplasms of the upper two-thirds of the rectum. Moreover, all patients underwent preoperative staging by total body CT with a three-phase contrast medium so that they could be accurately staged (according to the American Joint of Committee on Cancer 8th Edition) (18).

A possible limitation of this study may stem from the fact that this was a retrospective and non-randomized study, and the patients were assigned to two groups: the first group included patients who received a Knight–Griffen transanal termino-terminal anastomosis in which the section was sutured with a linear suturing machine-type ENDO-GIA and the second group included patients who received a transanal termino-terminal anastomosis in which a tobacco bag was placed on the rectal stump.

The variables analyzed were the duration of the postoperative stays in terms of days, the average time of the first postoperative flatus, and postoperative complications (AL, bleeding, perforation, occlusion, and infections).

In all patients, the surgical technique was standardized with respect to oncological radicality by ligating the artery and mesenteric vein at one centimeter from the origin to ensure a good local staging. Then, standardization was achieved by removing the nearby lymph node chain and freeing the pelvic rectum by performing a total mesorectal excision (TME), which is considered a guideline for rectal tumors.

In this study, the No-Coil tube was placed in all patients after the performance of intestinal anastomosis. It was placed through the sphincter and fixed at a 1–2 cm distance from the anus by making two dots; the tube was removed on the sixth postoperative day if there were no signs of AL. The No-Coil tube, mainly made of silicone, has a length of 60–80 mm, a thickness of 2 mm, and a diameter of 20 mm.

Non-severe surgical complications were corrected by using conservative treatment (Grades I and II according to Clavien Dindo Classification) (19).

Only one exclusion criterion was stipulated in this study: none of the patients should have been administered neoadjuvant therapy. Informed consent was obtained from all patients for performing the study and for the use of medical records. All procedures included in the protocol met the ethical standards of the Helsinki Declaration and the Guidelines for Good Clinical Practice.

## Results

Statistical analyses were performed using STATA version 14. Descriptive statistics comprised frequencies and percentages and means and standard deviations. Differences between the case and the control group patients were subsequently explored by using the *χ*^2^ test for categorical variables and the *T*-test for continuous variables. Furthermore, we performed a stepwise linear regression to establish the association between hospital stay (dependent variable), type of surgery, PPOI, AL events (independent variables), and No-Coil placement. All results obtained from the descriptive analysis and comparison between the two groups, called the “Knight–Griffen group” and the “TAPSSA group” (Trans-Anal Purse-String Suture Anastomosis), respectively, for a level of significance of 5% are presented in Table 1.

**Table 1:** Analysis and comparison between the two groups.

	TAPSSA (*N *= 38)	Knight–Griffen (*N* = 39)	*χ*^2^/*t*	*p*
Age	73.6 ± 5.4	71.6 ± 7.9	19.28	0.628
Gender				
Male	41		0.1225	0.122
Female	36			
Hospital stays	12.02 ± 1.8	6.89 ± 1.6		<0.001
POI	3.76 ± 1.7	3.07 ± 1.3	20.670	0.342
PPOI	9 (23.6%)	1 (2.56%)	7.597	0.006
AL	1 (2.63%)	0	1.039	0.308

**Figure 4:**
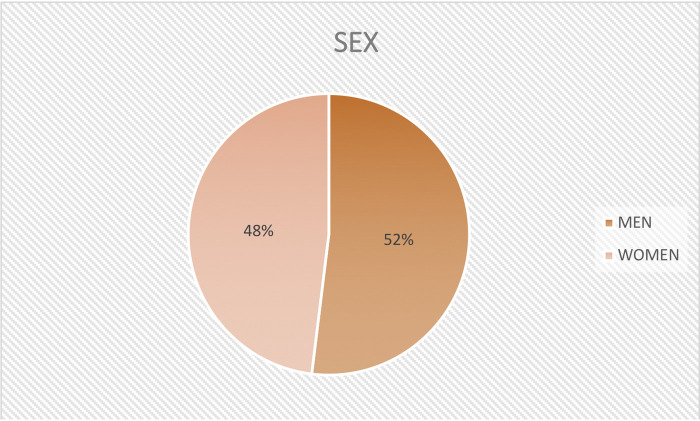
Percentage of men and women in the study.

No differences emerged in terms of gender (*χ*^2^ = 0.1225; *p* = 0.726) and age (*χ*^2^ = 19.3; *p* = 0.628) distribution between the two groups (Figure 4).

LC and LAR in LS were performed in 39 patients (50.65%) and LC and LAR by the open technique was performed in 38 patients (49.35%).

**Figure 5:**
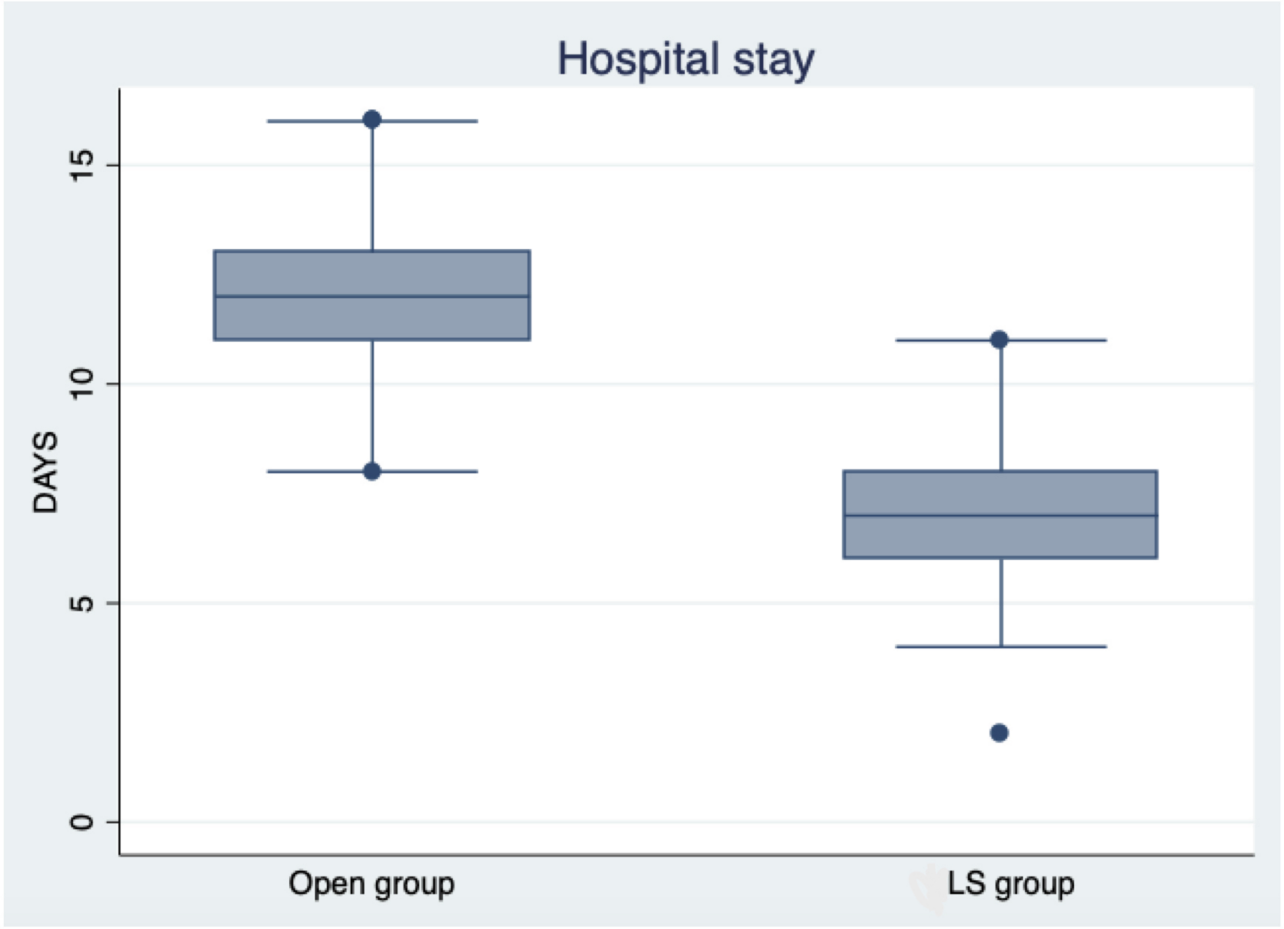
Box plot shows the difference between the open group and the LS group in terms of hospital stay.

No postoperative incontinence or constipation was reported in the patients. Mean hospital stay was 7 ± 1.6 days in LS group patients and 12 ± 1.8 in open group ones (Figure 5). AL was present in one patient (2.63%) in the open surgery group and in none in the LS group; AL in one patient was treated with the conservative method with total parenteral nutrition and removal of the No-Coil transanal tube on the 12th day. No statistical difference was detected in AL appearance between the groups (*χ*^2^ = 1.03; *p* = 0.308). PPOI was not associated with any independent variables. AL was not associated with POI days (log-likelihood = 5.0702; LR *χ*^2^ = 0.53; Prob > *χ*^2^ = 0.464; Pseudo *R*^2^ = 0.050;95% CI = 6.5269–14.271; *p* = 0.528) and also with PPOI (log-likelihood = 25.053; LR *χ*^2^ = 9.36; Prob > *χ*^2^ = 0.464; Pseudo *R*^2^ = 0.022; 95% CI = 2.266–3.227; *p* = 0.408).

A comparison of the results of the two groups showed that No-Coil placement reduced the postoperative complications of AL and POI with no differences between the two surgical techniques. But in terms of PPOI and hospital stay, the LS approach with Knight–Griffen anastomoses was found to be better than the traditional open methods with TAPSSA (Table 1).

## Discussion

The purpose of this retrospective study was to examine the differences between two types of surgery in terms of postoperative complications, AL, PPOI, POI, and hospital stay with the aid of the No-Coil® device. For the staging of the disease, particularly for the detection of liver and extrahepatic metastasis, we performed a full-body CT scan, which, according to different studies, is considered the first-choice procedure and the best imaging examination in terms of cost-effectiveness (20, 21). In this study, only one out of a total of 77 patients suffered AL. Hence, no differences were detected on the basis of the use of No-Coil after the performance of the surgery. Only a few studies have explored the efficacy of No-Coil use in reducing this type of leak. In one of them, Montemurro et al. evaluated AL prevalence in a sample of 184 patients undergoing elective total or subtotal proctectomy with low-lying anastomosis and found an AL incidence rate of almost 4.8%, which was higher than that of this study (14). Particularly, two randomized trials considered the use of a transanal stent other than No-Coil structurally different from it. Sciuto et al., in their study, analyzed the predictive factors of anastomotic losses after laparoscopic colorectal surgery, and there were several studies between 2008 and 2018 on laparoscopic colorectal procedures with left anastomosis (7).

In these studies, the incidence rates ranged from 78% (Lee et al.) in 2017 to 50% (Van Praagh et al*.*) in 2016 with an average of 11.1% (22, 23).

Considering all the above factors and that in our study we had only one patient with AL, which may be attributed to the placement of No-Coil, further follow-up studies are needed so that more insights can be had about AL (15). Amin et al. analyzed the occurrence of AL following the placement of an LAR plus transanal stent, as opposed to Tau protein (defunctioning stoma), and found anastomotic leakage in 3 of 41 patients (approximately 7%) (9). In contrast, Bulow et al*.* found that the transanal stent was not superior to a defunctioning stoma in preventing the risk of AL after LAR (about 10.7%) (11). Chen et al., who studied 1,262 patients without, and 1,170 patients with, a transanal drainage tube after laparoscopic anterior resection for rectal cancer concluded that placement was associated with significantly lower rates of AL and reoperation, and hence, it was likely to be an effective method of preventing and reducing AL after rectal cancer surgery (24). Zhao et al.*,* in 576 consecutive patients, indicated that the transanal drainage tube may not confer any benefit for AL prevention in patients who undergo laparoscopic low anterior resection for mid-low rectal cancer without preoperative radiotherapy (25).

Although contrary opinions are expressed in studies in the literature, almost all focus on a single technique and complication using different types of devices. These study results cannot be compared with the present results because the efficacy of different devices varies depending on the different purposes for which they are used. The device used by us is also a unique and non-fungible one, and the results obtained by us are related more to postoperative complications (AL, PPOI, POI, hospital stay) and two different patient groups that were compared, in which two different techniques were used, the Knight–Griffen group with the LS approach vs. the TAPSSA group with the traditional open method.

Our data are preliminary in nature and may have prevented us in finding the significance between the two different techniques in terms of AL and POI postoperative complications. For these reasons, other studies are necessary to confirm the evidence presented. The mean age of the patients of the groups was approximately 70 years and not lower.

This study confirmed, as shown in the literature, that laparoscopic intervention is characterized by a lower rate of PPOI than open surgery. In 13 RCTs instead, defining PPOI as a “reintegration of SNG”, the incidence rate of PPOI was 14.2% (95% CI 7.2%–26%) for colon or rectum resections and 30.9% (95% CI 12.7%–57.8%) for rectal resections. After laparoscopic resections, the incidence rate of PPOI was lower, approximately 6.4% (95% CI 3.5%–11.5%), but 10% (95% CI 6.2%–15.8%) after open colorectal resection (26, 27). Logically, it is not possible to compare these data with those of our study, as the definitions of PPOI, the number and characteristics of patients, and the type of intervention are not unambiguous in nature. Nevertheless, the fact that it was never necessary to reintroduce the SNG and that on the fifth postoperative day the incidence of PPOI was low suggests that the use of No-Coil has a positive influence on PPOI.

## Conclusion

According to the data collected on 77 patients, even in such a preliminary type of study, we can reasonably assume that with the positioning of the No-Coil tube, we were able to reduce the time of canalization to gases and feces and the risk of AL in both groups. Furthermore, by associating the No-Coil placement with the LS approach, we can reduce a number of postoperative complications related to PPOI and hospital stay. Another great advantage relating to the quality of life of the patients, their psychological health, and the costs for the National Health System stems from the elimination of the need to perform protective ileostomy and the subsequent stoma closure surgery. Also, we observed that the use of No-Coil allowed the patients to achieve faster mobility, given the high level of importance attached to the performance of these major surgical procedures.

## Data Availability

The raw data supporting the conclusions of this article will be made available by the authors without undue reservation.
